# Prosocial Behavior and Childhood Trajectories of Internalizing and Externalizing Problems: The Role of Neighborhood and School Contexts

**DOI:** 10.1037/dev0000076

**Published:** 2015-11-30

**Authors:** Eirini Flouri, Zahra Sarmadi

**Affiliations:** 1 Department of Psychology and Human Development, UCL Institute of Education, University College London

**Keywords:** emotional and behavioral problems, neighborhood effects, prosocial behavior, school effects

## Abstract

This study investigated the role of the interaction between prosocial behavior and contextual (school and neighborhood) risk in children’s trajectories of externalizing and internalizing problems at ages 3, 5, and 7. The sample was 9,850 Millennium Cohort Study families who lived in England when the cohort children were aged 3. Neighborhood context was captured by the proportion of subsidized (social rented) housing in the neighborhood and school context by school-level achievement. Even after adjustment for child- and family-level covariates, prosocial behavior was related both to lower levels of problem behavior at school entry and to its trajectory before and after. Neighborhood social housing was related to the trajectory of problem behavior, and school-level achievement to lower levels of problem behavior at school entry. The negative association between prosocial and problem behavior was stronger for children attending low-performing schools or living in disadvantaged neighborhoods. The adverse “effect” of low prosocial behavior, associated with low empathy and guilt and with constricted emotionality, on internalizing and externalizing problems appears to be exacerbated in high-risk contexts.

Prosociality is a trait that can be characterized by the tendency to care for, share with, and help other people. Prosocial behavior benefits others and/or promotes positive social relationships but is also related to subjective well-being in both children (Eisenberg, Fabes, & Spinrad, 2006) and adults (Weinstein & Ryan, 2010). Its relation with child mental health is more complex. Although prosocial behavior is negatively related to externalizing (“acting-out”) problems, it co-occurs with both high and low internalizing (anxiety and depressive) symptoms (Nantel-Vivier, Pihl, Côté, & Tremblay, 2014). It does appear, however, that engagement in prosocial behavior is linked to subsequent social information processing, an important determinant of children’s social adjustment (Crick & Dodge, 1994), such that children engaging in prosocial behavior show benign attribution biases and socially competent response strategies (Laible, McGinley, Carlo, Augustine, & Murphy, 2014; Nelson & Crick, 1999). Laible et al. (2014) showed that engaging in prosocial behavior likely evokes positive responses from others, which, in turn, cement children’s positive internal working models and trust in the goodness of others.

A trait facilitating social interactions, prosocial behavior in children is nonetheless unrelated to social (e.g., neighborhood or school) contexts (Romano, Tremblay, Boulerice, & Swisher, 2005), in turn important determinants of their emotional and behavioral outcomes (for a review, see van Ham & Manley, 2012). However, research has not yet examined if risky social contexts such as poor neighborhoods or disadvantaged schools may interact with prosocial behavior to predict problem behavior in children. This is a significant gap because there is evidence that adverse social contexts may accentuate the effects of individual vulnerabilities (Rutter, 1985).

## This Study

Using longitudinal data from a large British birth cohort followed from ages 3–7, we carried out this study to fill this gap. Our main aim was to examine the role of contextual (neighborhood and school) risk in moderating the association between children’s prosocial behavior and their trajectories of internalizing and externalizing problems. We focused on two contextual risk factors that previous research has identified as important for children at this age: percentage of subsidized housing in the neighborhood and school-level achievement. Subsidized housing (or “social housing”) accounts for about 18% of homes (Randall, 2011) in Great Britain. Neighborhoods with a high concentration of housing that is socially rented have high levels of crime, unemployment, antisocial behavior, and stigma and low levels of adult educational attainment and mental health (Lawder, Walsh, Kearns, & Livingston, 2014), all of which have been associated with problem behavior in children (Odgers et al., 2012). The effect of school-level achievement, on the other hand, on child well-being is less clear. It appears that school-level achievement is related negatively to academic and positively to behavioral outcomes. For example, Marsh and Hau (2003) have shown that it affects pupils’ academic self-concept negatively by enabling unfavorable social comparison processes. The relatively scarce research on its role in behavioral outcomes suggests that school-level achievement is related negatively to individual students’ internalizing and externalizing problems (Midouhas, Kuang, & Flouri, 2014).

## Method

### Sample

We used data from the Millennium Cohort Study (MCS; www.cls.ioe.ac.uk/mcs), a longitudinal survey of 19,244 families drawing its sample from all births in the United Kingdom over a year, from September 1, 2000. The MCS was designed to overrepresent areas with high proportions of ethnic minorities in England, areas of high child poverty, and the three smaller U.K. countries. Sweep 1 took place when the children were around 9 months. Sweeps 2, 3, and 4 (Times 1, 2 and 3, respectively), when internalizing and externalizing problems were measured, took place around ages 3, 5, and 7. We analyzed data from Times 1–3. We used records for only one child per family (the first-born where there were twins or triplets). Our analytic sample comprised children living in England (for which school-level performance data were available) at age 3 (*n* = 10,086) and with a score for internalizing or externalizing problems in at least one of Times 1–3 (*n* = 9,850). Complete data on internalizing and externalizing problems were not necessary because growth curve modeling, which we adopted, is able to handle unbalanced data (Snijders & Bosker, 1999).

### Measures

It is difficult to establish causal relationships between prosocial and problem behavior because many factors might jointly determine high levels of prosocial behavior and low levels of internalizing and externalizing problems. For example, more educated parents are more likely to have both well-adjusted (Midouhas et al., 2014) and prosocial (Newton, Laible, Carlo, Steele, & McGinley, 2014) children. We therefore controlled for maternal education. Because our main moderator variables were neighborhood social housing and school-level achievement, we also adjusted for family social rented housing and child cognitive ability to avoid attributing to neighborhoods and schools what are essentially family and child effects. To account for length of exposure to “risky” school and neighborhood contexts, we controlled for residential mobility (home moves between all four sweeps), too. Finally, in view of our outcome, we adjusted for maternal psychological distress and the child-level covariates of sex and ethnicity. Girls, in general, are at lower risk of behavioral problems than boys (Egger & Angold, 2006). The main ethnic minority groups in the United Kingdom have similar or better mental health than White British children for common disorders and higher rates for some less common conditions (Goodman, Patel, & Leon, 2008). The following describes how the key study variables were measured. All variables, unless otherwise specified, were measured at each time point, that is, ages 3, 5, and 7.

Internalizing and externalizing problems were measured with the parent-reported Strengths and Difficulties Questionnaire (SDQ; Goodman, 1997) subscales of emotional symptoms, hyperactivity/inattention, conduct problems, and peer problems. Each SDQ subscale has five items (scored from 0 = *not true* to 2 = *certainly true*), and the four subscale scores are added to compute a “total difficulties” score, measuring overall level of problem behavior. The SDQ offers the following mapping:

Internalizing: emotional symptoms (“Often complains of headaches”; “Many worries”; “Often unhappy, downhearted”; “Nervous or clingy in new situations”; “Many fears, easily scared”) and peer problems (“Rather solitary, tends to play alone”; “Has at least one good friend*”; “Generally liked by other children*”; “Picked on or bullied”; “Gets on better with adults than with other children”)
Externalizing: hyperactivity (“Restless, overactive”; “Constantly fidgeting or squirming”; “Easily distracted, concentration wanders”; “Doesn’t think things out before acting”; “Sees tasks through to the end*”) and conduct problems (“Often has temper tantrums or hot tempers”; “Generally disobedient”; “Often fights with other children”; “Often lies or cheats”; “Steals from home, school or elsewhere”)

In our sample, internal consistency was at acceptable levels (see Table 1) and in line with other SDQ research (Stone, Otten, Engels, Vermulst, & Janssens, 2010).[Table-anchor tbl1]

Prosocial behavior was measured with the SDQ’s prosocial behavior scale, also completed by parents, of five items scored 0–2. The items are as follows: “Considerate of other people’s feelings”; “Shares with other children”; “Helpful if someone is hurt, upset, or ill”; “Kind to younger children”; and “Volunteers to help others.”

Neighborhood social housing was measured with the percentage (from the 2001 U.K. Census) of adults living in social housing in the neighborhood (i.e., lower super output area [LSOA]), banded into quintiles. LSOAs cover around 1,500 inhabitants, with boundaries drawn to maximize social homogeneity. They are built from groups of Census Output Areas (typically 4–6) and are constrained by the boundaries of the Standard Table wards used for 2001 census outputs.

School-level achievement was measured as the achievement of schools attended by MCS children at Time 2 (around age 5). This was assessed with the school-level Key Stage I (KS1)^1^ average point scores of state-maintained schools, collected during the January 2006 school census. The KS1 data were banded into deciles. KS1 assessments are not administered to pupils until the end of Year 2 of school. Therefore, these KS1 data apply to a different cohort of children than the MCS children. However, we chose to measure the achievement of schools attended by MCS children at around age 5, when children in England start school full-time, to align with our measurement of the neighborhood social housing “effect” at age 5 (see Analytic Strategy).

The child-level covariates were sex, ethnicity, and nonverbal cognitive ability. Nonverbal cognitive ability was measured at age 5 with the Pattern Construction subscale of the British Ability Scales II (Elliott, Smith, & McCulloch, 1996), assessing children’s nonverbal reasoning. This test is very similar to the Block Design task from the Wechsler Preschool and Primary Scale of Intelligence–Revised (Wechsler, 1989), but it contains a larger range of items (two-, four-, and nine-block patterns) and can be used for a wider age range (3–17 years). The child constructs a design by putting together flat squares or solid cubes with black and yellow patterns on each side. The test yields a composite score, based on both accuracy and speed. The family-level covariates were maternal education, maternal psychological distress, social rented housing, and residential mobility. Maternal education was the mother’s highest academic qualification by Time 3. Maternal psychological distress was measured with the six-item Kessler scale (Kessler et al., 2003), which assesses the experience of recent, nonspecific psychological distress (α = .86–.88 across the three time points). Social rented housing was a binary dummy of whether the child’s family lived in social housing. Residential mobility was a binary indicator of whether the family changed address between sweeps.

### Analytic Strategy

First, we investigated whether the families in our analytic sample (*n* = 9,850) were different from those not in it (*n* = 236) on our study variables. Then we inspected the correlations between total difficulties, prosocial behavior, neighborhood social housing, and school-level achievement. Finally, we fitted three-level growth curve models to allow for the hierarchical nature of our data and avoid the underestimation of standard errors. The data set has repeated measures (at ages 3, 5, and 7) of total difficulties (Level 1) nested in children (Level 2) nested in schools at age 5 (Level 3). In all, in the original sample of 10,086 children, the school ID was missing for 1,309. The total number of schools was 3,331. Each school had 1–30 MCS children. Schools with only one MCS child (2,026 schools) were included because they contribute to the estimates of individual-level characteristics in the fixed-effects part of the model, even though they do not contribute to the variance between schools. All conditional models (Models 2–3) adjusted for area stratum to reflect the stratified sampling design of MCS, an approach that replaces the use of survey weights. We specified a random slope on the child’s age to allow for changes in problems across time to vary between children.

The full sequence of models estimated is as follows. Model 1 (unconditional model) investigated the average levels and growth of problems by regressing them on age in years (grand mean centered at age 5.06 years) and its square (as the average trajectory was U-shaped; see below). Grand mean centering age at the “midpoint” minimizes the correlation between age and age^2^, thus stabilizing the estimates (Raudenbush & Bryk, 2002). Model 2 added prosocial behavior, school-level achievement, and neighborhood (LSOA) social housing, all specified to be related to the intercept and (linear and quadratic) slopes of total difficulties. It also added the child and family covariates. Model 3 added the interactions between prosocial behavior and school-level achievement and between prosocial behavior and neighborhood social housing on total difficulties and their trajectory. All models were fitted in MLwiN 2.28 (http://www.bristol.ac.uk/cmm/software/mlwin/).

## Results

### Bias Analysis and Descriptives

As expected, the families in our analytic sample were more advantaged than those in the nonanalytic sample, lived in less deprived neighborhoods, and sent their children to higher performing schools. The mothers in the analytic sample had more education and less psychological distress than those in the nonanalytic sample, and their children had higher nonverbal cognitive ability. However, the two groups differed in prosocial behavior only at the last time point (age 7), with children in the analytic sample scoring higher than those excluded from it (tables available on request). Based on the correlations (see Table 1), there was evidence for the expected negative interrelationship between prosocial behavior and total difficulties. Also as expected, school-level achievement and neighborhood social housing were related to total difficulties but were unrelated (or very weakly related) to prosocial behavior.

### Models

The average trajectory of total difficulties was U-shaped. All random effects were statistically significant with the most variation found between children at central age and within children. In Model 2 (see Table 2), prosocial behavior was related to both total difficulties at age 5 and their trajectory before and after. School-level achievement was negatively correlated with total difficulties (but not their trajectory), and neighborhood social housing was related to the trajectory of total difficulties only. Model 3 showed that the effect of prosocial behavior on total difficulties at age 5 was moderated by neighborhood social housing, and its effect on the trajectory of total difficulties was moderated by school-level achievement.^2^

**Figure 1 fig1:**
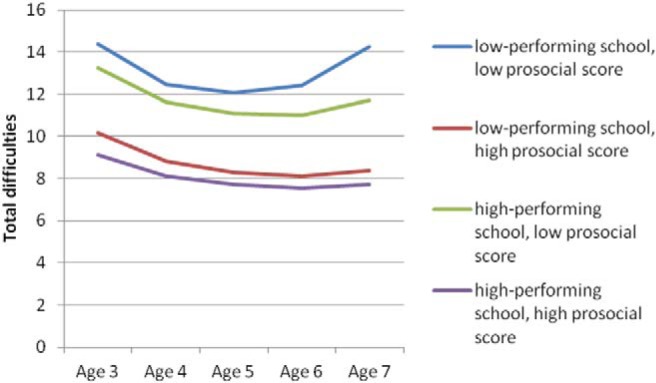
Predicted trajectories of total difficulties by prosocial behavior for children in high- and low-performing schools (Model 3). “Low-performing school” is the bottom decile of schools based on school-level Key Stage I average point scores, and “high-performing school” is the top decile. “High prosocial score” is the top decile of prosocial behavior score, and “low prosocial score” is the bottom decile. Predictions are plotted for the reference group for each categorical variable and at the mean of each continuous variable. See the online article for the color version of this figure.

**Figure 2 fig2:**
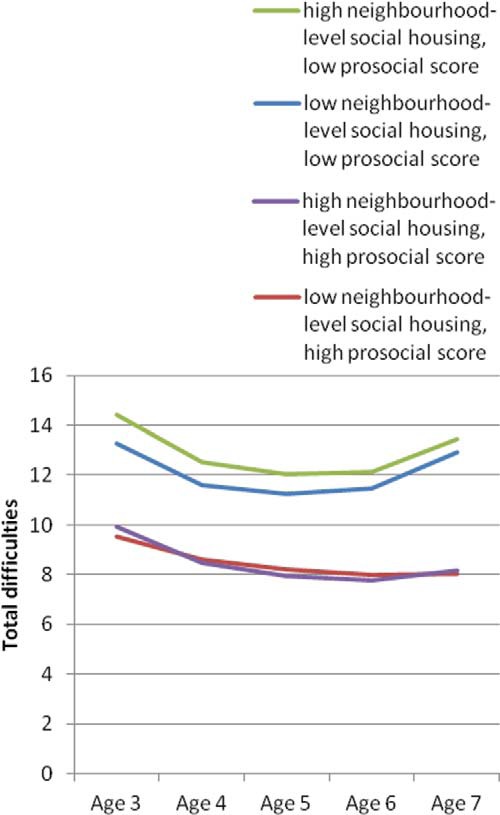
Predicted trajectories of total difficulties by prosocial behavior for children in neighborhoods with high and low proportions of social renters (Model 3). “Low neighborhood-level social housing” is the bottom quintile of neighborhoods based on the proportion of adult residents in social rented housing, and “high neighborhood-level social housing” is the top quintile. “High prosocial score” is the top decile of prosocial behavior score, and “low prosocial score” is the bottom decile. Predictions are plotted for the reference group for each categorical variable and at the mean of each continuous variable. See the online article for the color version of this figure.

Figures 1 and 2 plot these interaction effects. As can be seen, high levels of prosocial behavior were related to low levels of total difficulties, irrespective of neighborhood disadvantage or school-level achievement. The adverse effect of low prosocial behavior, however, was exacerbated at high levels of contextual risk and was particularly strong in the older ages among those in low-performing schools.[Fig-anchor fig1][Fig-anchor fig2]

## Discussion

Previous research has shown that prosocial activities both predict a range of adolescent health outcomes and interact with contextual risk to predict those outcomes (for a review, see Xue, Zimmerman, & Caldwell, 2007). We carried out this study to investigate the role of contextual (neighborhood and school) risk in moderating the longitudinal association between prosocial and problem behavior in early childhood. Following children from the United Kingdom’s MCS from ages 3–7, we found that when prosocial behavior—a constellation of behaviors characterized by cooperation, caring, and empathy—was low, children had poor emotional and behavioral outcomes across the study period. Importantly, the sizable adverse effect of low prosociality was exacerbated in both the high-risk contexts we considered (i.e., disadvantaged neighborhoods and low-performing schools). These high-risk contexts, in turn, were associated with problem behavior at school entry or its trajectory, even after bias due to selection was accounted for and other relevant parent and child covariates were controlled. The effects of these contexts were small compared to those of prosocial behavior or other risk factors of problem behavior such as maternal psychological distress. Nevertheless, both contexts modified the effect of prosocial behavior on problem behavior. High levels of prosocial behavior were related to low levels of total difficulties, irrespective of neighborhood disadvantage or school-level achievement. However, the adverse effect of low prosocial behavior was exacerbated at high levels of contextual risk. Children who were low in prosocial behavior and attended low-performing schools were on a high problem behavior trajectory, and children who were low in prosocial behavior had significantly more internalizing and externalizing problems at school entry if they lived in disadvantaged neighborhoods.

These cross-level interactions are of great substantive interest because they help identify with precision which children in which contexts may be prioritized for intervention. There is already evidence that school-based interventions to promote prosocial behavior help reduce aggression (Caprara et al., 2014). Our findings suggest that such interventions may be even more effective in low-performing schools. They also suggest, in line with previous findings (Biglan & Hinds, 2009), the importance of engaging children in prosocial activities, especially in disadvantaged neighborhoods or low-performing schools. Of course, this suggestion is predicated on the (as yet untestable in MCS) assumption that prosocial behavior, as we measured it, is related to prosocial activities. However, even if this assumption turns out not to be correct, our findings suggest that children with low prosociality in disadvantaged contexts are a particularly high-risk group for internalizing and externalizing problems. Nonetheless, we must caution against another assumption: that children with high prosociality are a uniformly low-risk group. As others have shown (Nantel-Vivier et al., 2014), the relationship between prosociality and child mental health is complex, with both very low and very high prosocial behavior conferring risk (as proxies for callousness and overconcern for others, respectively) for behavior and mental health difficulties in children.

Our findings must be viewed in the light of two important study limitations. The reliance on the mother’s reports of her mental health and her child’s prosocial behavior and internalizing/externalizing problems means that correlations between these measures are likely inflated by shared respondent variance. Related to this, the use of the SDQ to measure both prosocial and problem behavior means that shared measurement effects could have increased associations between the behavioral difficulties and prosocial behavior scales. Despite these limitations, our study has many strengths. This was the first study to examine how prosocial behavior and contextual risk interact to predict children’s trajectories of internalizing and externalizing problems. Its additional strengths are the use of a large, nationally representative cohort of children followed from early to middle childhood, as well as the simultaneous examination of both neighborhood and school contexts. This examination confirmed that disadvantaged contexts such as poor neighborhoods and low-performing schools have relatively modest effects on problem behavior in the early years. Neighborhood disadvantage, measured as proportion of subsidized housing in the neighborhood, had an effect on the trajectory of internalizing and externalizing problems, and school-average achievement was negatively related to individual children’s internalizing and externalizing problems at the beginning of primary school, but effects were small. However, both disadvantaged neighborhoods and low-performing schools exacerbated the effect of low prosocial behavior, an already robust risk factor of problem behavior in children.

## Figures and Tables

**Table 1 tbl1:** Correlations Among Neighborhood Social Housing, School-Level Achievement, Total Difficulties, and Prosocial Behavior

Variable	1	2	3	4	5	6	7	8	9	10
1. Neighborhood social housing, 3 years (*n* = 9,849)										
2. Neighborhood social housing, 5 years (*n* = 8,778)	.889**									
3. Neighborhood social housing, 7 years (*n* = 8,067)	.819**	.904**								
4. School-level achievement, 5 years (*n* = 8,134)	−.365**	−.377**	−.360**							
5. Total difficulties, 3 years (α = .64) (*n* = 9,137)	.196**	.193**	.189**	−.200**						
6. Total difficulties, 5 years (α = .67) (*n* = 8,441)	.171**	.170**	.178**	−.194**	.611**					
7. Total difficulties, 7 years (α = .69) (*n* = 7,856)	.169**	.169**	.171**	−.185**	.546**	.691**				
8. Prosocial behavior, 3 years (α = .66) (*n* = 9,266)	−.010	−.002	−.014	.019	−.355**	−.202**	−.191**			
9. Prosocial behavior, 5 years (α = .67) (*n* = 8,533)	−.032**	−.027*	−.031**	.065**	−.277**	−.398**	−.306**	.415**		
10. Prosocial behavior, 7 years (α = .70) (*n* = 7,921)	−.036**	−.024*	−.036**	.059**	−.265**	−.318**	−.416**	.357**	.522**	
*M*	3.314	3.242	3.180	5.341	9.857	7.417	7.636	7.328	8.361	8.568
*SD*	1.395	1.403	1.410	2.845	5.369	4.999	5.446	1.903	1.683	1.648
*Note.* Cronbach’s alphas for scales and observed number for all items in parentheses.										
* *p* < .05. ** *p* < .01.										

**Table 2:** Fixed Effects Estimates (Unstandardized Regression Coefficients and Standard Errors) and Variance Covariance Estimates of Growth in Total Difficulties (Model 2)

	Coefficient (*SE*)
Fixed effects	
Stratum (ref = England-advantaged)	
England-disadvantaged	.165 (.118)
England-ethnic	−.077 (.199)
Age	.114 (.110)
Age^2^	.397** (.077)
Girl	−.493** (.097)
Child’s ethnicity (ref = White)	
Mixed	−.170 (.259)
Indian	.462 (.303)
Pakistani/Bangladeshi	.808** (.238)
Black	−.629** (.271)
Other	.766 (.453)
Nonverbal cognitive ability	−.059** (.005)
Residential mobility	.358** (.079)
Maternal education (ref = No qualification)	
Higher degree	−2.348** (.281)
First degree	−2.453** (.205)
A level or HE diploma	−1.740** (.179)
GCSE_a-c_	−1.290** (.156)
GCSE_d-g_	−.520** (.189)
Other	−.519 (.318)
Maternal psychological distress	.253** (.013)
Maternal psychological distress × age	−.002 (.005)
Maternal psychological distress × age^2^	.010** (.003)
School-level achievement	−.084** (.024)
School-level achievement × age	.001 (.007)
School-level achievement × age^2^	−.005 (.005)
Neighborhood social housing	.019 (.045)
Neighborhood social housing × age	−.034* (.015)
Neighborhood social housing × age^2^	.021* (.010)
Social rented housing	.688** (.143)
Social rented housing × age	.036 (.049)
Social rented housing × age^2^	−.025 (.031)
Prosocial behavior	−.730** (.029)
Prosocial behavior × age	−.036** (.011)
Prosocial behavior × age^2^	−.030** (.007)
Constant	17.220** (.425)
Random effects	
Level 3 (school) intercept	.138 (.113)
Level 2 (child) intercept	9.707** (.273)
Slope	.424** (.035)
Covariance	.150* (.060)
Level 1 (occasion) intercept	7.594** (.161)
*Note.* HE = higher education; GCSE = General Certificate of Secondary Education.	
* *p* < .05. ** *p* < .01.	

## Footnotes

1 Key stages are stages of the state education system in England. KS1 applies to ages 5–7 (Years 1–2). Children are assessed in English, math, and science at the end of KS1. KS1 data are only available for state-maintained schools, and therefore these data were missing for those MCS children not attending state schools (around 8% of our sample).

2 Given that the total number of missing data points was about 8%, we also carried out a multiple imputation to deal with missingness. We created 10 multiply imputed data sets, using REALCOM-Impute within MLwiN. The results (available on request) remained largely unchanged. The following four variables became significant: England-disadvantaged, Indian, neighborhood social housing, and “other” maternal qualification.

* Inversely coded.
